# Blocking TGF-*β* Expression Attenuates Tumor Growth in Lung Cancers, Potentially Mediated by Skewing Development of Neutrophils

**DOI:** 10.1155/2022/3447185

**Published:** 2022-04-21

**Authors:** Yifei Fang, Xiyao Li, Yuxuan Jiang, Zhihan Ge

**Affiliations:** ^1^Faculty of Art and Science, University of Toronto (St. George Campus), Toronto, Ontario, Canada M5S 1A1; ^2^Nanjing University, Nanjing, Jiangsu 210033, China; ^3^Qingdao Academy, Qingdao, Shandong 266114, China; ^4^College of Life Science and Technology, Changchun University of Science and Technology, Changchun, Jilin 130013, China

## Abstract

In the tumor microenvironment (TME), cells secrete a cytokine known as transforming growth factor-*β* (TGF-*β*), which polarizes tumor-associated neutrophils (TANs) towards a protumor phenotype. In this work, C57BL/6 mice with *TGF-β1* gene knocked out selectively in myofibroblasts receive orthotopic implantation of Lewis lung carcinoma (LLC). Then, TANs' differentiation and tumor growth are studied both *in vivo* and *in vitro*, to examine the potential effects of TGF-*β* levels in TME on neutrophil polarization and cancer progression. Possible results are anticipated and discussed from various aspects. Though tumor suppression via inhibition of TGF-*β* signaling has been widely studied in this field, this study is the first to present a detailed experimental design for evaluating the potential antitumor effects of blocking TGF-*β* expression. This work provides a creative approach for cancer treatment targeting specific cytokines, and the experimental design presented here may apply to future research on other cytokines, promoting the development of novel cancer-treating strategies.

## 1. Introduction

### 1.1. NSCLC: Lethal Diseases That Lack Effective Diagnosis and Treatment Strategies

Nonsmall cell lung cancer (NSCLC) is a potentially lethal disease, whose incidence is rising rapidly worldwide, especially in the past 50 years. In 2020, there were about 19.3 million new cancer cases and 10.0 million deaths worldwide, with lung cancer accounting for 11.4% of new cases and 18% of deaths, making it the leading cause of cancer death [1]. Apart from the high incidence, the severity of NSCLC may be attributed to the difficulty in its diagnosis. The asymptomatic early stages of NSCLC cause the tumors in almost two-thirds of patients to develop into the advanced stages even before diagnosis.

To attenuate tumor growth, patients with NSCLC are usually treated surgically and postoperative chemotherapy and/or radiotherapy. However, patients with advanced lung cancer are not eligible for surgery, while conventional chemotherapy and radiotherapy do not increase patients' survival rate effectively and have severe adverse effects. To improve NSCLC patients' survival and quality of life, new strategies for NSCLC treatment are in urgent need.

It is worth noticing that NSCLC tumor cells produce multiple inflammatory cytokines, which recruit inflammatory cells such as neutrophils to the tumor microenvironment (TME) and activate them to promote cancer progression [2]. The recruited immune cells and the cytokines that activate them may be potential targets for cancer treatment. This study will be focusing on the tumor-associated neutrophils (TANs) and the transforming growth factor-*β* (TGF-*β*), a cytokine that is believed to influence the phenotypes of TANs. By exploring the effect of blocking the production of TGF-*β* on neutrophils differentiation and, therefore, cancer progression, this study hereby proposes a novel hypothetical treatment approach.

### 1.2. Vital Roles of Neutrophils in the Tumor Microenvironment

Neutrophils play a key role in the immune system and polarize into different phenotypes in response to environmental signals [3]. They facilitate adaptive immunity by contributing to B lymphocyte maturation via effector molecules and cytokines including BAFF and APRIL [4]. In addition, neutrophils mediate innate immune destruction of invading microorganisms through phagocytosis, releasing lyase, and producing reactive oxygen-species (ROS) [4]. They also release the neutrophils extracellular traps (NETs) that disarm pathogens. These extracellular fibrillary networks mainly consist of DNA and antimicrobial proteins, which kill microbes extracellularly and prevent further spread of pathogens [5].

Neutrophils play dual roles in the development of tumors, depending on their phenotypes and effector molecules produced [6]. To distinguish subsets of TANs, neutrophils with antitumor activities are known as N1 and the protumorigenic neutrophils are known as N2 [6]. N1 and N2 have different influences on TME, both directly and indirectly through the recruitment and activation of other cells. N1 neutrophils are capable of killing cancer cells by releasing of reactive oxygen species and reactive nitrogen species, as well as activating cytotoxic T cells and recruiting proinflammatory macrophages [7]. In contrast, N2 neutrophils promote tumor growth by inhibiting natural killer cell function and releasing matrix metalloproteinase 9 (MMP9), which stimulates angiogenesis and dissemination of cancer cells [7]. N2 neutrophils also recruit anti-inflammatory macrophages and regulatory T cells, which further facilitate cancer progression.

The role of TANs in NSCLC has not been extensively studied. However, during the treatment of patients with NSCLC, the neutrophil to lymphocyte ratio (NLR) is a commonly used prognostic marker of immunotherapy [8]. This suggests that neutrophils may have important effects on the progression of NSCLC and can be a potential target for NSCLC treatment.

### 1.3. TGF-*β*: Functions and Origins

The phenotypic switch in TANs is thought to be regulated by TGF-*β*, which is the most well-studied cytokine in the TGF-*β* superfamily. As a multifunctional cytokine, TGF-*β* has a great influence on the inflammatory response, bone remodeling, and cell differentiation. More importantly, it has been shown that TGF-*β* contributes to the growth of tumor cells, which makes it a potentially suitable target for cancer treatment. One of the major ways by which TGF-*β* stimulates tumor growth is that it induces N2 polarization of TANs, which inhibits the antitumor function of T cell and NK cells [9]. It has been demonstrated that blocking TGF-*β* activity inhibits the progression of the colorectal cancer (CRC) via the polarization of TANs towards N1 [10]. However, the antitumor effect of inhibiting TGF-*β* has not yet been tested in NSCLC.

In TME, tumors can promote TGF-*β* production through different pathways. For instance, TC-1 and B16-OVA tumor cells can secrete TGF-*β* in order to suppress the immunological function of plasmacytoid dendritic cells (pDC) [11]. Tumor cells can stimulate platelets to release large amounts of TGF-*β* to assist metastasis [12], and osteoclasts during tumor bone metastasis produce a large amount of TGF-*β* [13]. Thus, identifying and blocking the source of the TGF-*β* surge in TME would be a viable cancer treatment option. Hence, the authors hypothesize that blocking the expression of TGF-*β in vivo* will attenuate tumor growth in NSCLC, potentially mediated by skewing development of TANs' subpopulations.

Several research papers that have led to the novel immunotherapeutic approach to treat NSCLC are described in the following section. The chosen papers have provided evidence for the significance of the antitumor neutrophils subpopulation in resistance against selected cancers, as well as the vital roles of TGF-*β* in the polarization of neutrophils towards protumor phenotypes. The papers have also demonstrated that inhibiting TGF-*β* activities leads to tumor suppression, which has provided the foundation for the hypothetic treatment for NSCLC by blocking the expression of TGF-*β*.

## 2. Approach

### 2.1. Summary of Primary Research

#### 2.1.1. Function of Neutrophils in Tumor Microenvironment

In [7], neutrophils are a key player in the tumor microenvironment and are considered to be associated with cancer progression. By performing both bulk and single-cell RNA sequencing (scRNA-seq) assays on gene knockout mice, the researchers demonstrated that neutrophils are required for the activation of an interferon-gamma-dependent pathway of immune resistance, mediated by the polarization of a subset of CD4^−^CD8^−^ unconventional *αβ* T cells (UTC*αβ*). In selected human tumors, the researchers found that neutrophil infiltration was associated with a type 1 immune response and better clinical outcome. To conclude, these experiments showed the importance of neutrophils in resistance against murine sarcomas and selected human tumors.

#### 2.1.2. Anti-TGF-*β* Inhibits Cancer Progression via the Polarization of TANs to an anti-Tumor Phenotype

The [10] investigated the role of anti-TGF-*β* on the polarization of TANs towards a tumor-suppressive phenotype. Firstly, the researchers found that patients with colorectal cancer (CRC) showed higher TANs' infiltration and increased levels of TGF-*β* compared to the control. To further evaluate the roles of TANs and TGF-*β* in TME, SW480 cells established from a primary adenocarcinoma of the colon were cultured *in vitro* with primed neutrophils, which can be considered as TANs. Anti-TGF-*β* was added to block TGF-*β* in order to polarize TANs. The addition of anti-TGF-*β* not only suppressed the tumor migration by decreasing the metastasis chemoattractant produced by TANs but also promoted the apoptosis of cancer cells by increasing the cytotoxicity of TANs. This altered phenotype of TANs was potentially due to increased GM-CSF and INF-*γ* expression, which are cytokines that regulate the polarization of TANs. Further immunoblotting showed that the tumor-suppressive effect was mediated by the inhibition of PI3K/AKT signaling pathways in TANs and TGF-*β*/Smad signaling pathways in the tumor cells. Lastly, to explore the tumor-suppressive effect of anti-TGF-*β in vivo*, mice models were treated with anti-TGF-*β*. The tumors in the treated mice were significantly smaller and showed reverse tumorigenesis. To conclude, this study provided evidence that inhibiting TGF-*β* by anti-TGF-*β* could attenuate cancer progression via the polarization of TANs towards an antitumor phenotype, providing novel ways to cancer treatment.

#### 2.1.3. Integrin Subunit *α*_V_ Expressed by Tumor Cells Activates TGF-*β*

The [19] study showed that cancer cells express an integrin subunit known as *α*_V_, which activates TGF-*β* in the tumor microenvironment (TME). When first secreted, TGF-*β* is bound to latency associated protein and has no effector functions. The activation of TGF-*β* reshapes TME by polarizing the neutrophils towards a protumor phenotype, helping the tumor cells to evade the attack of the immune systems. Additionally, the inhibition of TGF-*β* maturation via *α*_V_ knockout promotes the differentiation of activated cytotoxic T cells to granzyme B-producing CD103+CD69+ resident memory T cells, which induce apoptosis in tumor cells. To conclude, this study demonstrated how the tumor cells evade the immune responses via TGF-*β* activation, suggesting that TGF-*β* may be a good target for cancer treatment.

#### 2.1.4. Finding and Inhibiting the Origin of TGF-*β* in TME

In [13], the authors revealed the role of TGF-*β* in the differences in the effectiveness of immune checkpoint therapy (ICT) in subcutaneous and skeletal castration-resistant prostate cancer (CRPC) models. They discovered that Th17 cells without antitumor function replace antitumor Th1 cells in the presence of abnormally high levels of TGF-*β*, which is closely related to Th17 differentiation. Next, they hypothesized the TGF-*β* surge results from the overabundance of osteoclastic cells in bone metastases. To further support the hypothesis, the writers blockaded the osteoclast differentiation and activated factor NF-*κ*B, significantly suppressing the osteoclasts in the femur. Consequently, TGF-*β* levels were significantly reduced while no significant changes in other cytokine levels were observed. Thus, they identified the main source of TGF-*β* overabundance in bone metastases as the osteoclast cells. Lastly, the authors found that the survival rate of bone metastatic CPRC mice was significantly enhanced after anti-TGF-*β* injection, and increased levels of CD8+ Tc, a marker for ICT, were detected, confirming that blocking TGF-*β* can be an effective way to strengthen the effect of ICT. Considering TGF-*β* is a vital protumor cytokine, the ideas provided in this paper for discovering and inhibiting the origin of TGF-*β* in the tumor microenvironment may have great potential in the treatment of other tumors.

The studies discussed above have demonstrated the importance of TGF-*β* in the polarization of TANs towards tumor-suppressive phenotypes, which have been identified as a key player in the immunity against cancers. The activity of TGF-*β* may also be inhibited to suppress tumor by skewing the development of TANs subsets. Combining these findings, the authors of this work hypothesize that inhibiting the expression of TGF-*β in vivo* assists in NSCLC treatment, potentially via the polarization of TANs' subpopulations. The general approach and detailed experiment designs are discussed in the next section.

### 2.2. Method and Materials

#### 2.2.1. General Approach

A series of experiments will be performed on four groups of C57BL/6 mice. The treatment group is mice with the *TGF-β1* gene knocked out specifically in myofibroblasts, showing low production of TGF-*β* in TME. One control group is wild type, healthy mice with normal level of TGF-*β* in TME. The second control group represents mice with TGF-*β* overexpression and consists of wild type mice with frequent injections of purified TGF-*β* to keep the level of TGF-*β* in TME high. The last group is comprised of mice treated with 1D11, which is the monoclonal antibody specific to TGF-*β*. The blockade of TGF-*β* has been shown to polarize TANs towards N1 and induce antitumor response [10]; thus, this group of mice is set up to examine the effectiveness of *TGF-β1* gene knockout therapy compared to the known antitumor effect of direct TGF-*β* inhibition.

The level of TGF-*β* in TME will be measured, and their N1 and N2 will be quantified to show how TGF-*β* level affects TANs' differentiation. The progression of cancer in each group will be examined by studying tumor phenotypes and marker expression. In addition, four media will be prepared, each of which will contain Lewis lung carcinoma cells, primed neutrophils, and myofibroblasts obtained directly from a specific group of mice model. The media allow the experiments to be repeated *in vitro*, so that it can be more confidently concluded that the findings of this work solely result from the change in TGF-*β* level in TME.

#### 2.2.2. Mice Model and Animal Care

Pathogen-free C57BL/6 mice with mixed genders at 6 weeks old will be used in this study. All mice will have free access to a standard laboratory diet and water *ad libitum*. The mice will be kept under controlled temperature and a 12 h light and dark cycle. The experimental procedures will be performed based on institutional animal care guidelines.

#### 2.2.3. Tumor Cell Line

Lewis lung carcinoma (LLC) cell line originated from mouse lung will be used in this study. LLC cells will be maintained as monolayer cultures in Dulbecco's Modified Eagle Medium (DMEM) with 10% fetal bovine serum (FBS) and penicillin/streptomycin and kept within 5% CO_2_ chamber and under 37 degrees Celsius.

#### 2.2.4. Orthotopic Intrapulmonary Implantation of LLC

C57BL/6 mice will be anesthetized with ether before surgery. A limited skin incision to the left chest with a length of approximately 5 mm will be made to each mouse, and 3 × 10^4^ LLC cells will be suspended in PBS buffer and orthotopically injected into the lung parenchyma. After injection, the skin incision will be closed by a surgical skin clip. Throughout the implantation procedure, the vital signs of mice including respiration rate and heart rate will be monitored. The transplanted tumors are allowed to develop for two weeks before the mice are sacrificed by euthanasia.

#### 2.2.5. Tissue Specific Knock-out Mice via Cre-loxP System

C57BL/6 mice that are homozygous for a *TGF-β1* gene flanked by *loxP* sites will mate with C57BL/6 mice that are hemizygous for myofibroblast-specific *cre* transgene and homozygous for *TGF-β1* genes that are not floxed. The cross will generate mice that are heterozygous for the floxed allele and hemizygous for the *cre* transgene. The F1 generation will then mate with the homozygous floxed mice. One fourth of the offspring will be homozygous for the floxed allele and hemizygous for the *cre* transgene, and they will be the *TGF-β1* knock-out mice used in further experiments. The *cre* transgenic mice without any *loxP*-flanked alleles will have normal TGF-*β* expression and will be one of the control groups. If the *Cre-loxP* system fails, then CRISPR-Cas9 will be used as an alternative gene editing tool.

#### 2.2.6. Tissue-Specific Gene Knockout via CRISPR-Cas9

Single guide RNA (sgRNA) will be designed via GenScript, whose algorithm is developed and validated by Feng Zhang lab, Broad Institute of Harvard and MIT. A px330 plasmid coding Cas9, sgRNA, and a myofibroblast-specific promoter will be introduced to fertilized, one-celled oocytes of C57BL/6 mice. The oocytes will then be transferred to pseudopregnant females. In this way, C57BL/6 mice with *TGF-β1* gene specifically knocked out in myofibroblasts will be generated via a Nonhomologous End Joining (NHEJ) approach.

#### 2.2.7. Direct Inhibition of TGF-*β* via Monoclonal Antibody 1D11

TGF-*β* antibody 1D11 (R&D Systems, Inc., MN, USA) is a highly potent TGF-*β* inhibitor that simultaneously inhibits TGF-*β*1, TGF-*β*2, and TGF-*β*3, with the most significant inhibitory effect on TGF-*β*1 signaling [14]. One of the four groups of mice will be treated with 1D11 three times per week, i.p, 5 mg/Kg to inhibit TGF-*β* signaling *in vivo*. To inhibit TGF-*β* signaling in the TME *in vitro*, one of the four media will be prepared by coculturing LLC cells, primed neutrophils, and myofibroblasts from wild-type mice in the presence of 1D11.

#### 2.2.8. LLC Conditioned Medium

LLC cells will be plated under the conditions described in the “*Tumor cell line*” section. When the cells are 50% confluent, the medium will be replaced by DMEM with 1% FBS. After 48 hours, the conditioned medium will be collected and used for neutrophils priming.

#### 2.2.9. Purification of Neutrophils

Around 10 mL of blood will be obtained from healthy, wild-type C57BL/6 mice and anticoagulated with heparin. The neutrophils will be isolated by density centrifugation, and their viability will be checked via trypan blue exclusion. The purity of neutrophils may be checked by Wright staining of cytocentrifuge slides.

#### 2.2.10. Neutrophil Priming

Before being cocultured with LLC cells in the presence or absence of 1D11, the purified neutrophils need to be primed by culturing in the conditioned medium of LLC cells for 6 hours. The primed neutrophils will adjust to the tumor microenvironment and may be considered as TANs in subsequent *in vitro* assays.

#### 2.2.11. Enzyme-Linked Immunosorbent Assay (ELISA)

This assay employs a quantitative sandwich enzyme immunoassay technique, using AssayMax Mouse TGF-*β*1 ELISA (Enzyme-Linked Immunosorbent Assay) kit to detect TGF-*β*1 in the supernatants of cell lysate and the plasma. TGF-*β*1 molecules in the standard solutions and the samples will be fixed to the plate by the immobilized antibody. Then, they will be bound by biotinylated polyclonal antibodies specific to mouse TGF-*β*1, which can be recognized by a streptavidin-peroxidase conjugate. All the unbound substances are then washed away, and a peroxidase enzyme substrate is added for the color to develop. The color development will be stopped and the absorbance at a certain wavelength will be measured. Concentrations of TGF-*β*1 will be calculated by comparing the absorbance with a standard curve generated by ELISA assays on TGF-*β*1 in standards.

#### 2.2.12. Measurement of Tumor Size and Weight

After the mice are sacrificed, the tumors will be removed by surgery. The weight of the tumors will then be measured, along with the length (longest dimension) and width (shortest dimension) of the tumors. The size of the tumor can then be calculated by the formula *V* = 1/2 (*L* × *W*)^2^ [15].

#### 2.2.13. Electrochemiluminescence Immunoassay (ECLI)

Tumor markers are molecules present in or produced by malignant cells or tumor-associated cells, and their upregulation often correlates with tumor growth. In this study, CEA, CA125, NSE, and cyfra21-1 are selected as the tumor markers for NSCLC, because increases in their serum concentrations are often detected in NSCLC patients. Prior to ECLI, blood will be collected from all groups of mice and centrifuged to obtain the serum. The level of the tumor markers in the serum will be quantified, by measuring their chemical electroluminescence. Elecsys1010 and kits provided by Roche in Germany will be used in the ECLI assay.

#### 2.2.14. Immunohistochemical Analysis

DAB staining will be performed on paraffin sections of lung tissues according to the instructions of DAB staining kit (Abcam). Antibodies specific to CD31 (Thermo Fisher) will be used in this study.

#### 2.2.15. Fluorescence-Activated Cell Sorting (FACS)

FACS will be performed on the cells in TME to quantify N1 and N2 neutrophils, which allows the researchers to study the effect of blocking TGF-*β* expression on TANs' polarization. The cells in TME will be harvested and washed to prepare a single cell suspension in ice cold FACS Buffer (PBS, 0.5-1% BSA or 5-10% FBS, 0.1% NaN_3_ sodium azide). To identify the TANs in the suspension, antibodies specific to LyG6 (Proteintech) will be added, which is expressed extensively on neutrophils [16]. To further distinguish N1 from N2 neutrophils, the cells will be labelled with antibodies specific to CD206 (Abcam), which is expressed on the surface of N2 but not N1. The two types of antibodies have unique fluorescent tags attached to them, so the labelled cells will emit light with different wavelengths when analyzed with a flow cytometer, allowing the identification of the distinct cell types.

## 3. Anticipated Results

### 3.1. TGF-*β* Expression in TME

**Figure 1:**
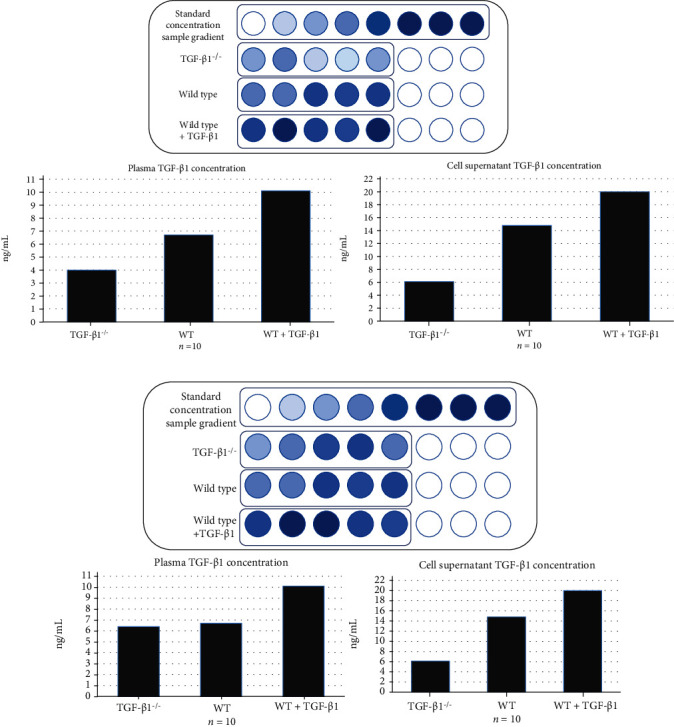
Predicted plasma TGF-*β*1 concentration and TGF-*β*1 concentration *in vitro* (a) Blood TGF-*β*1 concentration in TGF-*β*1 knockout mice is reduced compared with wild-type mice. In addition, the TGF-*β*1 concentration of mice with injection of purified TGF-*β*1 is significantly higher than that of wild-type mice. (b) Compared with the wild-type mice, there is no significant decrease in blood TGF-*β*1 concentration in the *TGF-β1* knockout group, indicating that the gene knockout model is not successful.

To study the efficiency of manipulating the expression of TGF-*β* in TME by specifically knocking out *TGF-β1* gene in mice myofibroblasts, the serum TGF- *β*1 level in mice will be quantified via ELISA. Additionally, the TGF-*β*1 level in the media containing purified LLC cells, TANs, and myofibroblasts will also be measured via ELISA. Since the level of TGF-*β* is not directly influenced in mice treated with 1D11 or in the medium prepared in the presence of 1D11, ELISA is not performed for these two groups of samples. The two possible results for the rest of the samples are shown in Figure 1 below.

### 3.2. Change in Tumor Growth in Response to TGF-*β* Level in TME

**Figure 2:**
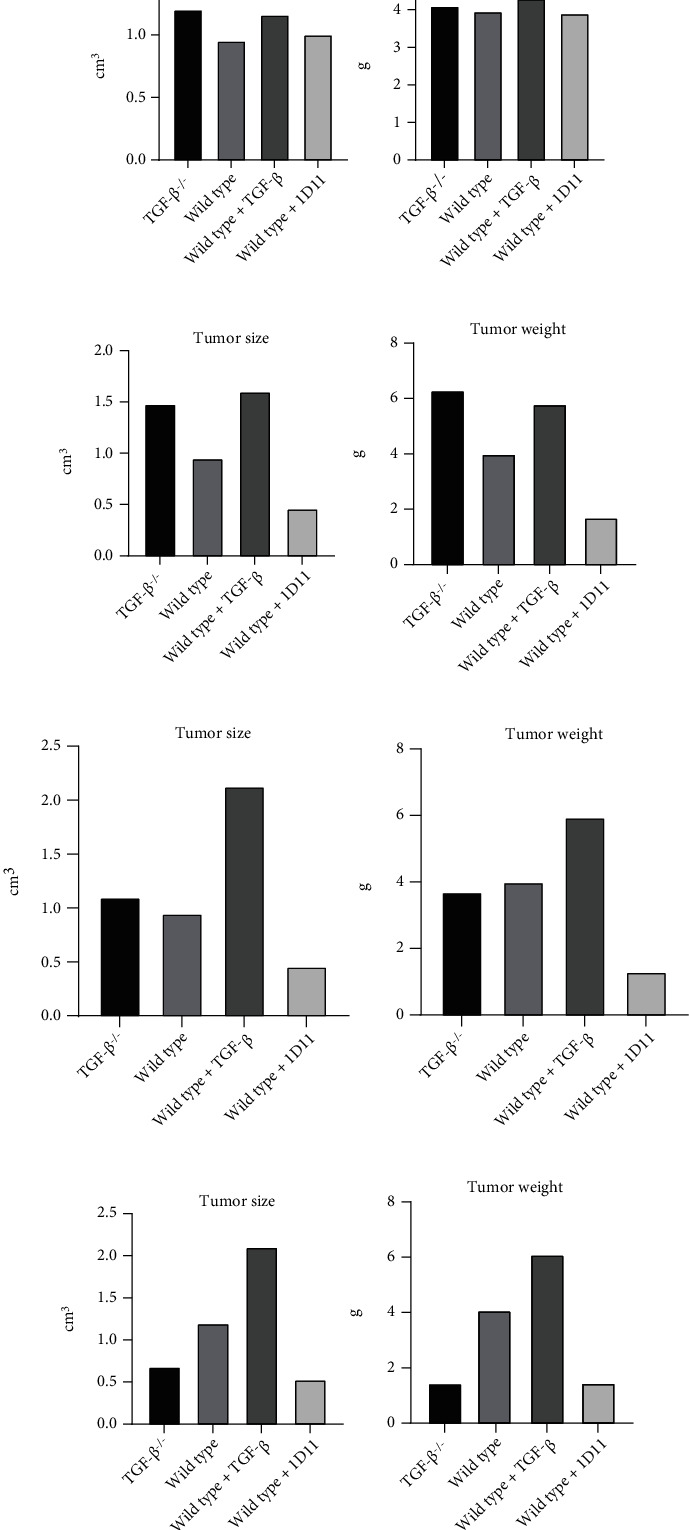
Predicted tumor size and weight (a) There is no significant difference in tumor growth among all mice. (b) *TGF-β1* gene knockout mice and mice injected with additional TGF-*β* develop larger and more severe tumors than wild-type mice, while the injection of 1D11 leads to reduced tumor growth. (c) There is no significant difference in tumor growth rate between the *TGF-β1* knockout group and the wild-type mice, while the tumors in mice injected with additional TGF-*β* are larger and heavier. The tumors in mice treated with 1D11 have reduced size and weight compared to those in wild-type mice. (d) In comparison to wild-type mice, the tumors in the gene knockout mice and 1D11-treated mice are smaller, while the tumors in mice treated with additional TGF-*β* develop more rapidly.

All mice will be sacrificed 2 weeks after orthotopic transplantation of NSCLC cells, and the size and weight of the NSCLC tumor will be measured. The four possible outcomes are shown in Figure 2.

### 3.3. Immunohistochemical Analysis

The lung tissues of mice will be sliced and stained with H and E and immunohistochemistry. It is expected that more blurred tumor margins and more microvascular infiltration around the tumor tissue are observed in mice with injections of additional TGF-*β*. In *TGF-β1* gene knockout mice and 1D11-treated mice, clearer tumor tissue margins, less microvascular infiltration, and fewer necrotic areas within the tumor sections are expected.

### 3.4. Detection of Tumor Markers

**Figure 3:**
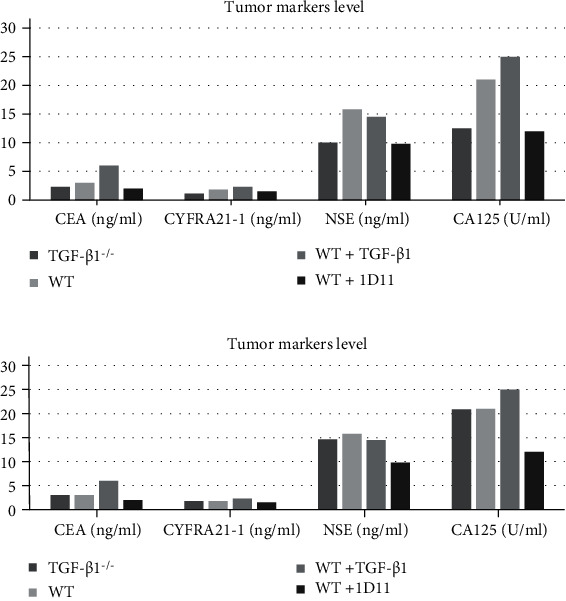
Predicted level of tumor markers in serum (a) Compared with wild-type mice, the concentrations of various tumor markers in both *TGF-β1* gene knockout mice and 1D11-treated mice decrease, while the concentrations of most tumor markers in the additional TGF-*β*1 injection group significantly increase. (b) Although additional TGF-*β*1 injections results in higher levels of most cancer markers, the gene knockout does not reduce the concentrations of tumor markers, suggesting that *TGF-β1* knockout therapy is ineffective.

The level of tumor markers CEA, CyFRA21-1, NSE, and CA125 in mice serum is measured to further study the progression of NSCLC under different TGF-*β* levels [17]. The two possible results are shown in Figure 3.

### 3.5. The Relative Abundance of N1 and N2 TANs in TME

**Figure 4:**
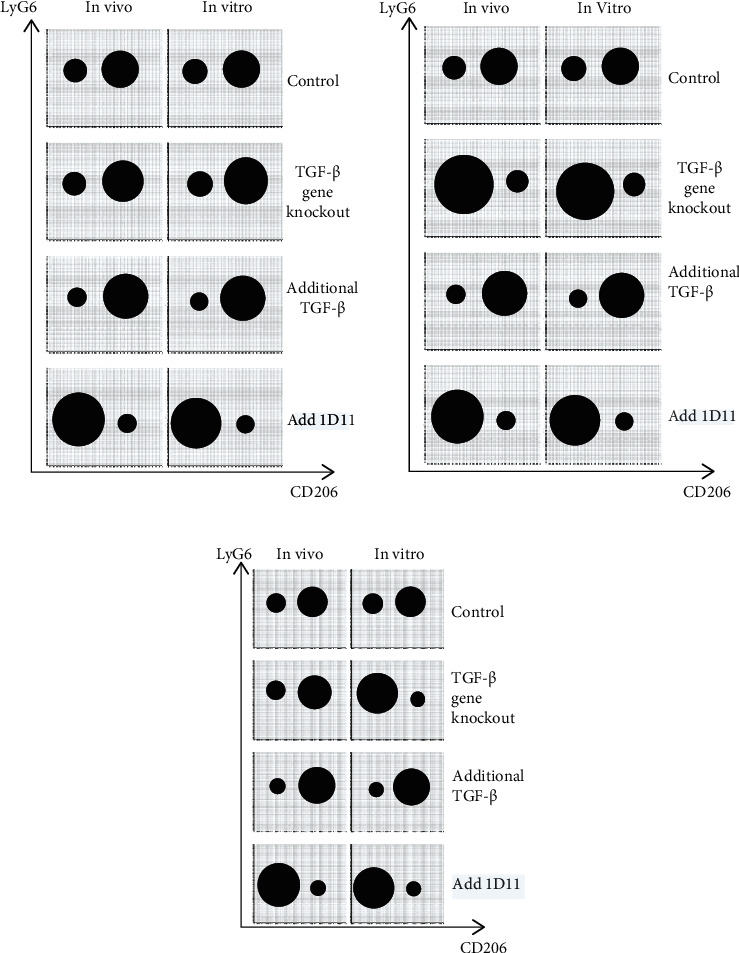
Predicted abundance of N1 TANs and N2 TANs quantified by flow cytometry. In each diagram, one solid black circle represents one population of cells, and the area of the circle correlates with the number of cells in that population. (a) Compared with the control group, the abundance of N1 (Ly6G+/CD206-) and N2 (Ly6G+/CD206+) cells in TME after gene knockout of TGF-*β* producing gene in myofibroblasts might not change significantly, and N2 (Ly6G+/CD206+) subgroup might be still more than the N1 (Ly6G+/CD206-) subgroup. After the addition of additional TGF based on the control group, the number of N2 (Ly6G+/CD206+) subgroup cells might further increase, and the number of N1 (Ly6G+/CD206-) subgroup cells might further decrease. The addition of 1D11 leads to increased N1 and decreased N2 population. (b) Compared with the control group, the number of N1 (Ly6G+/CD206-) subgroup significantly increases, and N2 (Ly6G+/CD206+) subgroup significantly decreases after the knockout of TGF-*β* producing gene in myofibroblasts. After the addition of additional TGF-*β* based on the control group, the number of N2 (Ly6G+/CD206+) subgroup cells might further increase, and the number of N1 (Ly6G+/CD206-) subgroup cells might further decrease. The addition of 1D11 results in expanded N1 and reduced N2. (c) There can be little difference between *in vivo* and *in vitro* controls. *In vivo*, compared with the control group, gene knockout of TGF-*β* producing gene in myofibroblasts might not affect the differentiation of N1 (Ly6G+/CD206-) and N2 (Ly6G+/CD206+). However, *in vitro*, gene knockout of TGF-*β* producing gene in myofibroblasts might result in a significant increase in the N1 (Ly6G+/CD206-) population and a significant decrease in the N2 (Ly6G+/CD206+) population. After the addition of additional TGF-*β* based on the control group, the number of N2 (Ly6G+/CD206+) subgroup cells might further increase, and the number of N1 (Ly6G+/CD206-) subgroup cells might decrease further. The addition of 1D11 promotes N1 and suppresses N2 differentiation.

To study the effect of inhibiting TGF-*β* production in myofibroblasts on the differentiation of TANs, FACS would be performed on the cells in TME to quantify N1 and N2 subpopulations. The anticipated results are shown in Figure 4.

## 4. Discussion

### 4.1. TGF-*β* Expression in the Tumor Microenvironment (TME)

The expected results are shown in Figure 1(a), where the level of TGF-*β* decreases in TME with *TGF-β1* knocked out myofibroblasts. This confirms that the tissue-specific gene knockout is successful, and myofibroblasts are a major source of TGF-*β*. In Figure 1(b), however, similar TGF-*β* levels are observed in the wild type and the *TGF-β1* knockout models, indicating that the gene knockout is not effective. Before any further assay, the tissue-specific gene knockout needs to be redone via CRISPR-Cas9. Alternatively, the ineffective knockout may be because myofibroblasts are not a significant source of TGF-*β* in TME. Therefore, in future experiments, *TGF-β1* may be knocked out in a different cell type or more than one cell type. Additionally, if the injection of purified TGF-*β* only leads to significantly higher TGF-*β* level *in vitro* but not *in vivo*, it is highly likely due to the catabolism of TGF-*β in vivo*, which may be compromised by more frequent injections of TGF-*β*.

### 4.2. Tumor Size and Weight

In Figure 2(a), no significant difference in the growth rate of tumors can be observed in four groups of models. In this case, TGF-*β* has little effect on tumor growth and the hypothesis is refuted. In Figure 2(b), *TGF-β1* knockout mice develop larger tumors than wild-type mice, while 1D11 treatment suppresses tumor growth. The reason behind the different outcomes between the two inhibition mechanisms may be that 1D11 only inhibits TGF-*β* in TME, while *TGF-β1* knockout blocks TGF-*β* expression in all myofibroblast cells throughout the body. Since TGF-*β*1 is a multifunctional cytokine widely present in the body, the knockout of *TGF-β1* may have an overall immune-suppressive effect, hence facilitating the development of cancer.  Figure 2(c) shows no significant difference in tumor growth rate between the *TGF-β1* knockout group and the wild-type mice, while mice with injections of TGF-*β* develop larger tumors. It is likely that the gene knockout in myofibroblasts does not effectively reduce the TGF-*β* level in TME. As previously discussed, the tissue-specific gene knockout method needs to be revised. Lastly, in Figure 2(d), compared to the wild-type mice, tumor growth in the *TGF-β1* knockout mice is attenuated and the mice with higher levels of TGF-*β* develop larger tumors. Thus, reducing the expression of TGF-*β* shows antitumor effects and the hypothesis is supported. The difference in the limitation of tumor growth between the *TGF-β1* knockout treatment and the 1D11 treatment also reflects the effectiveness of tumor suppression by blocking TGF-*β* production. However, the measurements of tumor size and weight only examine the growth of primary NSCLC tumors, while providing no information regarding tumor metastasis. Thus, follow-up experiments are required to study the effect of TGF-*β* level on tumor metastasis.

### 4.3. Immunohistochemical Analysis

More irregular tumor tissue boundaries and increased microvascular infiltration typically represent a more malignant tumor. Thus, if clearer tumor tissue margins and less microvascular infiltration are observed in the *TGF-β1* knockout mice compared to the wild type mice, then inhibition of TGF-*β* expression is shown to attenuate tumor growth, supporting the hypothesis. If the morphology of the tumors shows no appreciable differences among the mice models, the hypothesis might be refuted. However, the qualitative nature of tumor morphology makes the analysis inevitably subjective. Hence, the results need to be analyzed with quantitative data from other experiments in order to draw a more reliable conclusion.

### 4.4. The Level of Tumor Markers

Tumor markers such as CEA, CyFRA21-1, NSE and CA125 are commonly used in cancer diagnosis. Their abnormal upregulation often precedes clinical symptoms and other detection signals [18], so their concentrations may be measured to study the tumor growth. In Figure 3(a), the levels of most tumor markers decrease in mice with lower TGF-*β* expression, indicating that blocking the production of TGF-*β* in TME inhibits tumor growth, supporting the hypothesis. The effectiveness of *TGF-β1*-knockout therapy may be inferred by comparing the level of tumor markers in knockout mice and mice treated with 1D11, which has been shown to limit tumor growth via TGF-*β* inhibition [10]. In Figure 3(b), the levels of most tumor markers are higher in mice with TGF-*β* overexpression, demonstrating the protumor effects of TGF-*β*. However, the gene knockout does not reduce the expression of tumor markers, suggesting that *TGF-β1* knockout in myofibroblasts is not effective and CRISPR-Cas9 should be used as an alternative gene editing tool.

### 4.5. The Relative Quantity of N1 and N2 TANs in TME

In Figure 4(a), the differentiation of TANs into N1 and N2 both *in vivo* and *in vitro* is not significantly affected by the *TGF-β1* gene knockout of myofibroblasts, indicating that myofibroblasts may not be an important source of TGF-*β*. In future experiments, the *TGF-β1* gene may be knocked out in other types of cells to significantly reduce the amount of TGF-*β* in TME. If the decrease in TGF-*β* level still has no appreciable influence on neutrophils polarization, the hypothesis may be refuted. In Figure 4(b), both *in vivo* and *in vitro*, the tumor microenvironment (TME) with *TGF-β1* knocked out myofibroblasts contains increased amount of N1 and reduced amount of N2, compared to the control group. In TME with overexpression of TGF-*β*, however, the number of N1 decreases while the number of N2 increases. The results show a positive correlation between the TGF-*β* level and the polarization of TANs towards N2, which promotes tumor growth. Therefore, blocking the expression of TGF-*β* in TME shifts the differentiation of TANs towards an antitumor phenotype, supporting the hypothesis. As shown in Figure 4(c), the differentiation of TANs *in vivo* is not significantly affected by *TGF-β1* knockout, but more N1 and fewer N2 are observed in the *in vitro* media with *TGF-β1* knocked out myofibroblasts. The skewed differentiation observed *in vitro* indicates that myofibroblast is indeed a major source of TGF-*β* and that inhibiting TGF-*β* expression in TME polarizes TANs towards N1. However, there are more than one type of cells producing TGF-*β in vivo,* so the reduced production of TGF-*β* due to *TGF-β1* knocked out myofibroblasts may be compromised by other cells, leading to TANs' differentiation similar to the wild type models. In future experiments, *TGF-β1* may be knocked out in more TGF-*β* producing cell types. Under all three circumstances, the inhibition of TGF-*β* signaling via 1D11 is predicted to shift the differentiation of TANs towards antitumor (N1) phenotype, according to the results of Qin et al. [10].

### 4.6. Limitations

In this study, the production of TGF-*β* is selectively blocked in mice myofibroblasts, but not other tissues. The choice of myofibroblasts is based on the fact that myofibroblasts are one of the most abundant TGF-*β*-producing cells present in the NSCLC TME. Nonetheless, other cells like thrombocytes and tumor cells are also major contributors of TGF-*β* expression in TME. Hence, future experiments may be performed with selective *TGF-β1* knockout in a different type of cells or multiple types of cells. Another limitation is that the progression of cancer is only monitored via the growth of primary tumor, while the metastasis of NSCLC is not examined. Besides, the mechanism by which TGF-*β* contributes to the polarization of TANs is still a mystery. More in-depth research is in need to characterize the signaling pathway of TAN differentiation.

## 5. Conclusion

Tumor-associated neutrophils (TANs) significantly influence the progression of nonsmall cell lung cancer (NSCLC), and their effector functions are affected by a cytokine known as transforming growth factor-*β* (TGF-*β*), which polarizes the TANs towards a protumor phenotype. Thus, blocking the production of TGF-*β* may attenuate tumor growth through the polarization of TANs towards a tumor-suppressive phenotype. In this study, the C57BL/6 mice are divided into four groups, one with the *TGF-β1* gene knocked out in myofibroblasts, one with frequent injections of purified TGF-*β*, one containing wild type, healthy mice with normal expression of TGF-*β*, the other containing wild type mice treated with anti-TGF-*β* (1D11). After orthotopic intrapulmonary implantation of Lewis lung carcinoma (LLC), the mice's N1 and N2 in TME are quantified by flow cytometry to investigate to what extent TGF-*β* level influences TANs' differentiation. The morphology of the tumors and the level of tumor markers in serum are also examined to study the tumor growth under different TGF-*β* levels. The experiments are then repeated *in vitro* on media containing LLC cells, TANs, and myofibroblasts obtained from the mice model. Possible results are anticipated and discussed from various aspects. To conclude, this study provides experimental designs for studying the potential antitumor effects of blocking TGF-*β* production. Since most current studies in this field focus on the therapeutic potential of directly inhibiting TGF-*β* signaling, rather than blocking the production of TGF-*β* in TME, this study fills the knowledge gap and presents a creative direction for cancer therapy targeting specific cytokines. Combining with other cancer treatments, the tissue-specific blockade of TGF-*β* production may lead to promising outcomes and may have clinical applications. Furthermore, the experimental design presented in this study may apply to other cytokines, facilitating the development of novel cancer therapies.

## Data Availability

The data used to support the findings of this study are included within the article.
